# Newly Designed Optical Coherence Tomography Catheter for Optimizing Bladder Cancer Diagnosis and Treatment: Protocol for a Feasibility Study

**DOI:** 10.2196/76644

**Published:** 2025-12-02

**Authors:** Marinka Jolinde Remmelink, Jakko A Nieuwenhuijzen, Daniel Martijn de Bruin, Jorg R Oddens

**Affiliations:** 1 Department of Urology Amsterdam UMC Location University of Amsterdam Amsterdam The Netherlands; 2 Cancer Center Amsterdam Amsterdam The Netherlands; 3 Department of Urology Amsterdam UMC Location Vrije Universiteit Amsterdam Amsterdam The Netherlands; 4 Department of Biomedical Engineering and Physics Amsterdam UMC Location University of Amsterdam Amsterdam The Netherlands

**Keywords:** bladder cancer, optical coherence tomography, OCT, diagnosis, microelectromechanical systems

## Abstract

**Background:**

Bladder cancer diagnosis relies on cystoscopy and transurethral resection of bladder tumor (TURBT) for histopathological evaluation, but this process is time consuming, costly, and subject to variability. Optical coherence tomography (OCT) offers real-time, high-resolution imaging as a potential alternative.

**Objective:**

This study primarily aims to assess the feasibility of capturing in vivo cross-sectional images of the bladder wall using a novel microelectromechanical systems (MEMS)-based OCT catheter. Secondary objectives include evaluating measurement duration, assessing tumor stage and grade from OCT images in comparison with histopathology, determining the catheter’s ability to image resection beds, and comparing OCT-based tumor staging with white light cystoscopy assessments.

**Methods:**

This single-center feasibility study at Amsterdam University Medical Center includes patients undergoing TURBT for suspected bladder tumors. Eligible patients must be aged 18 years or older, have at least 1 cystoscopically accessible tumor, and must be physically fit for TURBT. Exclusion criteria include pregnancy, tumors larger than 2 cm, more than 5 tumors, isolated flat lesions, or tumors solely at the bladder neck. The primary end point assesses the procedural feasibility of OCT imaging, while the secondary end points evaluate tumor staging, grading, and correlation with histopathology. Up to 25 patients will be enrolled, with feasibility achieved if diagnostic images are obtained in more than 60% of the cases. OCT imaging is performed before and after tumor resection, with histopathological results used for comparison. Patients will be monitored for adverse events for 4 weeks after the procedure, after which study participation ends.

**Results:**

As of November 2025, 16 participants have been enrolled, and 13 have successfully completed the study procedure. The projected end date of the study is November 2025, and results are expected to be published in March 2026.

**Conclusions:**

This study is expected to provide key insights into the feasibility and clinical utility of the newly developed MEMS-based forward-looking OCT system for real-time bladder imaging during TURBT. This study will lay the groundwork for a larger trial to evaluate its diagnostic accuracy in staging and grading bladder cancer.

**Trial Registration:**

ClinicalTrials.gov NCT06679920; https://clinicaltrials.gov/study/NCT06679920

**International Registered Report Identifier (IRRID):**

DERR1-10.2196/76644

## Introduction

The current diagnostic process for bladder cancer involves a cystoscopy followed by transurethral resection of the bladder tumor (TURBT) [1]. A TURBT is undertaken to obtain material for histopathology, which is required to confirm the malignant nature of the lesion and, if so, to determine the invasion and grade of the tumor [1]. In some patients, a repeat TURBT is necessary to ensure complete resection and confirm the histopathological stage [1]. Determining the histopathological stage and grade is essential for an appropriate treatment plan. If the tumor is muscle invasive, the available treatment options are radical cystectomy or trimodality therapy [2]. Treatment of non–muscle-invasive bladder cancer comprises TURBT, followed by regular cystoscopy, and, depending on the differentiation grade of the tumor, additional Bacille Calmette-Guérin or chemotherapy instillations [1].

Although the histopathological result is currently regarded as the gold standard, there remains significant interobserver variability, which may have a considerable impact on treatment decisions [3]. Furthermore, the current diagnostic process places a substantial burden on patients with bladder cancer and results in substantial health care costs [4,5]. Both surgical planning and the histopathological process are time consuming as well. Therefore, there is a need for an alternative option to determine the tumor grade and stage in real time.

Such an alternative might be an “optical biopsy,” which uses an optical technique to ascertain the stage and grade of the tumor [6]. An optical biopsy can facilitate the diagnostic process and enable the implementation of alternative treatment options, such as outpatient laser fulguration or active surveillance [7,8]. One technique with the potential to perform such an “optical biopsy” is optical coherence tomography (OCT), which uses infrared light to generate high-resolution, real-time cross-sectional images of tissue [9,10]. OCT was first described in 1991 [11] and has since been used and studied in several tissue types, with encouraging results [12,13]. The OCT catheters typically have a helical-scanning and side-looking design, making them suitable for tubular structures, such as vessels and ureters, but unsuitable for other organs such as the bladder [14,15]. To facilitate the analysis of the bladder wall using OCT, it is necessary to use a forward-looking OCT catheter that has the ability to acquire OCT images within the field of view of the cystoscope.

Three distinct forward-looking OCT catheters have been investigated for use within the bladder in previous studies [16-21]. These studies reported varying results regarding the sensitivity and specificity of both catheters. While some findings were highly promising, others showed no advantage for OCT over white light cystoscopy (WLC). All the studied first-generation time-domain OCT systems used with the catheters had low frames-per-second rates, leading to distorted images and making them highly susceptible to motion artifacts. In addition, the one catheter previously available on the market had to be in contact with the tissue, resulting in a field of view that is only as large as the tip of the catheter (ie, 2.7 mm), limiting its practical use [17,18].

In light of the aforementioned considerations, we developed a novel forward-looking OCT catheter. Interfaced with a fast second-generation OCT system, this OCT catheter has a considerably higher frames per second, while using a wavelength of approximately 1060 nm, which minimizes infrared light absorption by water [22,23]. The new OCT catheter incorporates a microelectromechanical systems (MEMS) mirror combined with a graded index lens that has a focal plane at 10.9 mm (in air) from the OCT MEMS mirror, enabling the generation of an image with a larger field of view than the catheter tip diameter [24].

The primary objective of this study is to investigate the feasibility of capturing in vivo cross-sectional images of the bladder wall in patients with bladder cancer using the newly developed MEMS-based OCT catheter. Secondary objectives are to evaluate the duration of the OCT measurements, determine the tumor stage based on OCT images and test for an association with histopathological results, determine the grade of the tumor based on OCT images and test for an association with the histopathological results, determine if the OCT catheter is able to acquire evaluable images of the bladder wall after resection of the tumor, and compare the tumor grade and stage estimated during WLC with the tumor grade and stage based on the OCT images.

This is a single-group; idea, development, exploration, assessment, and long-term (IDEAL) phase 2a (development) [25]; single-center feasibility study.

## Methods

### Study Population and Setting

The study population comprises adult patients with a suspected bladder tumor, either non–muscle-invasive bladder cancer or muscle-invasive bladder cancer, planned for a TURBT. This study is performed at the Amsterdam University Medical Center (UMC). The trial has been registered on ClinicalTrials.gov (NCT06679920).

### Eligibility Criteria

The eligibility criteria are presented in Textbox 1.

**Inclusion and exclusion criteria.**

**Inclusion criteria**

- Aged older than 18 years
- Have at least 1 suspected bladder tumor seen on cystoscopy
- Have a bladder that is accessible for cystoscopy
- Be in a physical condition to undergo transurethral resection of the bladder tumor
- Must be fully informed about the study and should provide written informed consent before any study-related investigation or intervention

**Exclusion criteria**

- Pregnant or lactating women
- Only tumor site at the bladder neck
- Tumor larger than 2 cm in diameter
- More than 5 tumors present
- Only flat suspected lesions

### Study Procedures

#### Standard Procedure

**Figure 1:**
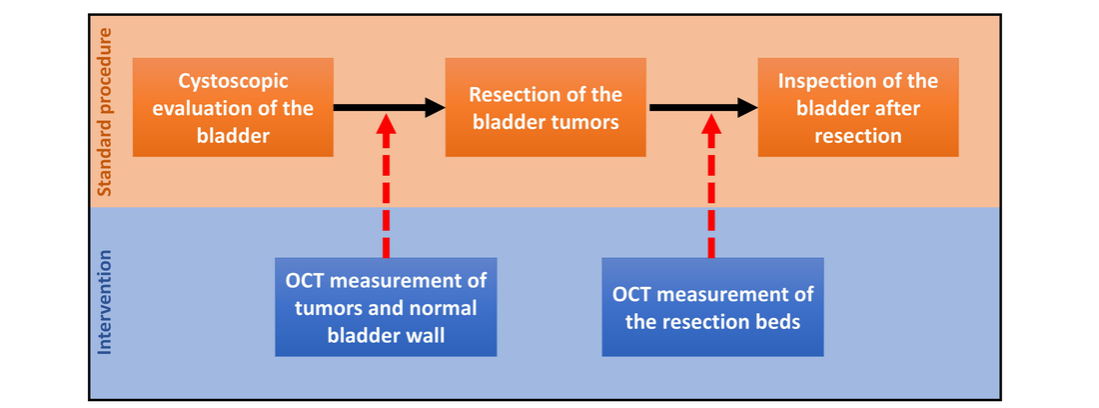
Schematic overview of the standard procedure and timing of the study interventions during transurethral resection of the bladder tumor.

The standard procedure is the TURBT. After regional or general anesthesia, a resectoscope is inserted into the bladder through the urethra. First, endoscopic inspection of the bladder wall is performed to identify tumors and suspicious lesions. Then, the identified tumors are resected using a loop wire with electric current. After resection of the tumor and retrieval of the resected tissue, the resection bed and edges are cauterized to ensure hemostasis. Subsequently, the resectoscope is removed, and, in most cases, a catheter will be inserted. Resected tissue is sent to pathology to determine the definitive stage and grade. Figure 1 provides an overview of the standard procedure and the schedule of the interventions.

#### OCT Measurements

During the standard TURBT procedure, the OCT measurements will be performed. After the inspection of the bladder and before the resection of the identified bladder tumors, the OCT catheter will be inserted through the working channel of the rigid resectoscope. The tumors will then be imaged with the OCT catheter tip at approximately 1 cm from the tumor. Approximately 5 measurements will be performed for each suspected lesion and tumor and 3 for a selected region of normal bladder wall. When the OCT measurements are finished, the OCT catheter is removed from the bladder, and the urologist will continue with the standard procedure. The resection of the tumors is performed according to standard care. However, the pathology specimens of each separate tumor will be collected and numbered according to the tumor numbers used during the OCT measurements and sent to the pathologist. After completion of the resection, the OCT catheter will be reinserted through the working channel of the rigid resectoscope, and approximately 3 OCT measurements will be performed on each resection bed. After these measurements, the OCT catheter will be removed from the bladder, and the urologist will continue with the standard procedure (Figure 1).

### Study Outcomes

#### Primary End Point

The primary end point comprises two components:

1. Procedural feasibility of OCT imaging will be determined by the ability to generate images of the normal bladder wall and bladder tumors, as assessed by the operating urologist. Procedural feasibility will be achieved if diagnostic images of the tumor and normal bladder are obtained in more than 60% of the patients.
2. Feasibility based on image use will be evaluated as the percentage of images suitable for diagnosis out of the total number of OCT images obtained. A percentage greater than 80% is regarded as feasible.

#### Secondary End Point

The secondary outcomes include (1) duration of the OCT measurements, (2) concordance between the OCT-based diagnosis of tumor invasion and histopathological diagnosis, (3) concordance between the OCT-based diagnosis of tumor grade and the histopathological diagnosis, (4) the ability of the OCT catheter to acquire evaluable images of the resection bed, and (5) concordance between intraoperative WLC assessments and OCT findings on tumor grade and invasion.

### Study Timeline

Patients will be identified at the outpatient clinic, and patients meeting the inclusion and exclusion criteria who provide informed consent will be scheduled for TURBT within 6 weeks of identification. The TURBT and the OCT catheter procedure will be performed as described previously. The procedural timeline of the TURBT is illustrated in Figure 1. After undergoing TURBT, participants will continue with standard treatment. Serious adverse events (AEs) will be monitored and recorded for a period of 4 weeks. Study participation will conclude at the end of this monitoring period.

### Sample Size and Enrollment

To date, no human study assessing the feasibility of this new MEMS-based OCT catheter for bladder cancer has been conducted. Therefore, a sample size calculation is not possible. The goal of this study is to test the technical and procedural feasibility of the device in a single-center, single-intervention, small prospective cohort study. In this IDEAL stage 2a study, up to 25 patients who undergo technically successful OCT measurements will be enrolled. Because the feasibility cutoff is set at 60%, inclusion will stop when a feasible procedure is performed in 15 patients. The findings of this study will inform sample size calculations for future investigations.

### Data Collection and Management

Data collection and analysis will be conducted in accordance with Good Clinical Practice guidelines to ensure data integrity and reliability. Outcome, baseline, and other trial-related data will be systematically collected from both paper-based and electronic sources, including case report forms completed during TURBT and electronic patient records. All data will be securely stored in Castor Electronic Data Capture, a validated electronic data capture system designed to prevent unauthorized access and ensure compliance with the General Data Protection Regulation. To maintain data completeness and allow for comprehensive analysis, all information collected up to the point of participant withdrawal or protocol deviation, including OCT measurements, cystoscopic video recordings, and histopathological findings, will be retained for subsequent evaluation.

To safeguard participant confidentiality, each enrolled individual will be assigned a unique participant ID code, with a corresponding participant ID code list maintained only when necessary for data traceability. The principal investigator will be responsible for securing the key to this code. In addition, individual OCT measurements will be assigned specific identifiers to ensure structured and organized data management. OCT data and cystoscopic video recordings will initially be stored on the OCT system and subsequently transferred to the local drive of the urology department on the secure Amsterdam UMC computer server using an encrypted external hard drive immediately after acquisition. Access to the local drive will be restricted to study investigators and the study monitor to ensure data security and confidentiality. Once the transfer is successfully completed, the data will be permanently deleted from the external hard drive. Upon completion of the study, all OCT images and cystoscopic video recordings will also be removed from the OCT system.

Robust data management protocols will be implemented to ensure data accuracy, security, and traceability. Data entry, coding, and security procedures will be conducted within Castor Electronic Data Capture, incorporating an audit trail to track all modifications. Paper-based case report forms will be securely stored, signed, and archived, while electronic data will be subject to automated validation procedures. Univariate data validations will be embedded within the Castor system, whereas multivariate validations, derivations, and calculations will be incorporated where applicable and documented in a dedicated data validation and derivation plan. The comprehensive data management strategy is outlined in this study’s data management plan. In accordance with regulatory requirements, all participant data will be securely retained for a period of 15 years.

Missing data will be descriptively reported and discussed. Missing OCT measurements will be reported as part of the main outcomes. Missing histopathological data are not expected; in case of missing data, it will be described in the secondary outcomes section.

### Statistical Methods

#### Primary Outcomes

Procedural feasibility will be achieved if images of both the tumor and normal bladder are obtained in more than 60% of the patients. The feasibility based on OCT imaging will be assessed by determining the percentage of images suitable for diagnosis out of the total number of images obtained in all patients. A panel, consisting of 2 urologists and 1 PhD candidate, blinded to clinical information, will individually analyze the OCT images. They will determine if the OCT images are suitable for diagnosis. The OCT measurements will be scored as suitable if (1) tissue is observed on the OCT image, (2) the tissue is within the focus range of the OCT, and (3) no movement artifacts are observed in the image. In case of disagreement, an experienced research fellow will be added to the panel, and the scoring will be discussed within this panel. Consensus will be reached when 3 of the 4 panel members agree. The data will be described and presented as percentages with 95% CIs. No statistical tests will be performed.

#### Secondary Outcomes

The duration of OCT measurements (min) will be reported as medians with IQR. Tumor invasion based on OCT images will be determined by a panel of investigators. The panel will consist of 2 urologists, 1 PhD candidate, and 1 pathologist. The panel members will independently score the OCT images and classify them based on (1) whether there is a tumor present and (2) (if present) the tumor stage. In case of disagreement, an experienced research fellow will be added to the panel, and the scoring will be discussed within this panel. Consensus will be reached when 3 of the 5 members agree. The concluding tumor invasions will be descriptively compared to histopathological outcomes, with results presented as percentages with 95% CIs. Attenuation coefficients from OCT data will be analyzed and categorized into tumor grade categories, following the World Health Organization (WHO) 1973 and WHO 2016 grading systems. A descriptive comparison of OCT-based and histopathology-based tumor grades will also be displayed as percentages with 95% CIs. Associations between tumor stage and grade, as determined by OCT and histopathology, will be tested with chi-square or Fisher exact tests, as applicable. In addition, the panel will assess OCT images of the resection beds, reporting the percentage of evaluable images with 95% CIs. Finally, the urologist’s WLC-based estimates of tumor invasion and grade will be compared to OCT findings and histopathological results, and the association will be tested with chi-square or Fisher exact tests, as applicable.

### Monitoring

#### Data Monitoring

Data monitoring will be conducted by the Clinical Monitoring Center of Amsterdam UMC. The clinical research associates of the Clinical Monitoring Center operate independently of both the study and the research team, ensuring unbiased oversight. The monitoring committee is responsible for verifying that the rights and well-being of participants are protected; the collected study data are accurate, complete, and verifiable against source documents; and the study is conducted in accordance with the approved protocol, applicable laws, and regulatory requirements. The study-specific monitoring schedule and procedures are documented in a signed monitoring plan.

#### Safety Criteria and Reporting

AEs will be reported according to the Common Terminology Criteria for Adverse Events (version 5.0). All AEs will be reported up to 4 weeks after the study procedure. Serious AEs will be reported to the ethics committee. Serious AEs that are related to the surgical procedure, without a likely relation to the study intervention, are reported through yearly line listing to the ethics committee. These events are bladder perforation, bladder infection, and hematuria requiring bladder catheterization.

### Ethical Considerations

#### Informed Consent

Patients will be informed about the study by their treating physician at the outpatient clinic. If interested, a second appointment will be planned with one of the investigators. All patients will be fully informed about the aims of this study, possible AEs, and the investigational procedure. Written informed consent is obtained from all participants before enrollment (refer to Multimedia Appendix 1 for the informed consent form). All physicians who may potentially see an eligible patient at the outpatient clinic are informed about the study procedures and criteria to ensure identification of potential participants.

#### Ethics Approval and Amendments

This study was approved on November 7, 2024, by the institutional review board of the Amsterdam UMC. All substantial amendments will be notified to the medical ethics review committee; nonsubstantial amendments will be recorded and filed by the sponsor. A summary of the trial progress will be submitted to the medical ethics review committee yearly, including information on the date of inclusion of the first participant, the number of participants included, the number of participants who have completed the trial, and serious AEs.

#### Provisions and Compensation

Provisions for ancillary and posttrial care are not applicable to this study design. The sponsor has insurance coverage for participants that provides compensation for injury or death directly attributable to the study.

## Results

As of November 2025, 16 participants have been enrolled, and 13 have already successfully completed the study procedure. The projected end date of this study is November 2025, and results are expected to be published in March 2026.

## Discussion

### Anticipated Findings

The anticipated principal finding of this study is that the novel MEMS-based forward-looking OCT catheter is safe to use and feasible for generating in vivo cross-sectional images of the bladder wall and tumor during TURBT, with most images suitable for interpretation. This IDEAL phase 2a study represents a critical step in evaluating cystoscopic OCT as a potential imaging tool to aid bladder cancer diagnosis and staging.

The principal findings of this feasibility study will provide critical insights into the utility of the new OCT system in visualizing bladder wall architecture and tumor invasion and grading. Furthermore, this study will provide insights into the clinical and technical obstacles that new devices face when being integrated into the diagnostic process of bladder cancer. In comparison with the current gold standard—histopathological evaluation following TURBT—OCT may enable more immediate, noninvasive diagnostic support, potentially streamlining treatment decision-making. Previously, 3 other forward-looking OCT probes have been studied on bladder tissue and showed that OCT is able to distinguish different layers of the bladder and, in most studies, was able to distinguish malignant tumors from benign tumors [16-21,26,27]. However, the previously studied OCT systems available for bladder wall imaging had, among other constraints, a limited field of view and a low frames-per-second rate, limiting their ease of use and the quality of the OCT images [16,21,27]. In contrast, our system has incorporated technical improvements, such as an improved OCT system with an operating wavelength centered at 1060 nm—which is optimal for imaging in water and forward-looking MEMS-based scanning mirror technology—that altogether enhance image acquisition, resolution, and usability. Importantly, this study will also explore secondary outcomes such as measurement duration and the ability to image resection beds.

One strength of the study lies in its integration into the routine clinical workflow without introducing significant treatment delays. However, this study also has important limitations. Most notably, no histopathological samples are taken from areas of the bladder deemed “normal.” This choice is deliberate and ethically justified; obtaining such biopsies would require additional surgical interventions that are not part of standard care and would therefore introduce unnecessary risks such as bladder perforation or bleeding. Instead, the assessment of the normal bladder wall will be based on the judgment of an experienced urologist during cystoscopy. Another limitation is the single-center design, which may limit the generalizability of the results because outcomes could be influenced by the urologist’s level of experience. However, this limitation is acceptable for an IDEAL phase 2a feasibility study.

Within the IDEAL framework, future studies will aim to validate these findings in larger, multicenter cohorts, with structured training modules for OCT image interpretation, and incorporate learning curves in the study outcomes. Further development may include the integration of artificial intelligence–based image analysis to support real-time diagnosis. In future research, correlation of OCT findings with clinical outcomes such as recurrence or progression could also enhance its clinical relevance. Nevertheless, for integration into clinical practice, the cost-effectiveness of OCT compared to TURBT remains an aspect that should be further studied.

The findings from this study will be published in open-access peer-reviewed journals and will be presented at urology and imaging conferences. In addition, results will be shared with clinical and research communities through institutional networks and multidisciplinary meetings, enabling translation into larger-scale clinical trials and potential clinical implementation if further validated.

### Conclusions

This trial aims to demonstrate the feasibility of using a newly designed MEMS-based OCT catheter and OCT system for the acquisition of diagnostic OCT images in human bladders. If the hypothesis is validated, it will lay the groundwork for a larger, multicenter trial to assess the diagnostic accuracy of OCT in staging and grading bladder cancer. The ultimate goal is to evaluate the potential role of this new OCT catheter and system as a real-time, noninvasive adjunct to standard cystoscopic assessment.

## Appendix

Multimedia Appendix 1 Informed consent form.

## Abbreviations

AE: adverse event
IDEAL: idea, development, exploration, assessment, and long-term study
MEMS: microelectromechanical systems
OCT: optical coherence tomography
AE: adverse event
TURBT: transurethral resection of the bladder tumor
UMC: University Medical Center
WHO: World Health Organization
WLC: white light cystoscopy

## Data Availability

Data sharing is not applicable to this study as no datasets were generated or analyzed during this study.
