# Supplementary material for: Newly Designed Optical Coherence Tomography Catheter for Optimizing Bladder Cancer Diagnosis and Treatment: Protocol for a Feasibility Study
Source: JMIR Res Protoc. 2025 Dec 2;14:e76644. doi: 10.2196/76644 (PMC12709158; doi:10.2196/76644)
Supplement: Multimedia Appendix 1 [file resprot_v14i1e76644_app1.pdf]

Appendix A. Informed Consent form

Proefpersoneninformatie voor deelname aan medisch-wetenschappelijk onderzoek

Nieuwe beeldvormende techniek voor blaastumoren tijdens blaaskijkonderzoek.

Officiële titel: IDEAL stage IIa studie van een nieuw ontworpen Optische Coherentie Tomografie katheter tijdens transurethrale resectie voor optimalisatie van blaaskanker diagnose en behandeling.

Inleiding

Geachte heer/mevrouw,

Met deze informatiebrief willen we u vragen of u wilt meedoen aan medisch-wetenschappelijk onderzoek. Meedoen is vrijwillig. U krijgt deze brief omdat u binnenkort een operatie ondergaat in verband met een tumor in de blaas. U leest hier om wat voor onderzoek het gaat, wat het voor u betekent, en wat de voordelen en nadelen zijn. Het is veel informatie. Wilt u de informatie doorlezen en beslissen of u wilt meedoen? Als u wilt meedoen, kunt u het formulier invullen dat u vindt in bijlage D.

Stel uw vragen

U kunt uw beslissing nemen met de informatie die u in deze informatiebrief vindt. Daarnaast raden we u aan om dit te doen:
- Stel vragen aan de onderzoeker die u deze informatie geeft.
- Praat met uw partner, familie of vrienden over dit onderzoek.
- Stel vragen aan de onafhankelijk deskundige. Voor contactgegevens zie bijlage A.
- Lees de informatie op www.rijksoverheid.nl/mensenonderzoek.

1. Algemene informatie

Het Amsterdam UMC heeft dit onderzoek opgezet. Hieronder noemen we het Amsterdam UMC steeds de ‘opdrachtgever’. Onderzoekers, dit kunnen ook artsen zijn, voeren het onderzoek uit in het Amsterdam UMC. Het onderzoek wordt gefinancierd vanuit de Europese Unie.
Deelnemers aan een medisch-wetenschappelijk onderzoek worden vaak proefpersonen genoemd. Zowel patiënten als mensen die gezond zijn, kunnen proefpersoon zijn.
Voor dit onderzoek zijn 15 proefpersonen nodig. De medisch-ethische toetsingscommissie van het Amsterdam UMC heeft dit onderzoek goedgekeurd.

2. Wat is het doel van het onderzoek?

In dit onderzoek bekijken we of de nieuwe optische coherentie tomografie (OCT) katheter een doorsnede afbeelding kan maken van blaastumoren en of op deze afbeeldingen de blaastumor beoordeeld kan worden. OCT maakt met behulp van infrarood licht een afbeelding van de verschillende lagen van de blaaswand.

3. Wat is de achtergrond van het onderzoek?

Voor het behandelen van blaaskanker is het belangrijk om te weten van welk type blaaskanker er sprake is. Het type wordt bepaald door de agressiviteit van de kankercellen en door de diepte van ingroei van de blaastumor in de blaaswand. Dit kan niet beoordeeld worden door middel van een kijkonderzoek in de blaas. Om te bepalen van wat voor tumor type er sprake is moet de uroloog de tumor met een operatie verwijderen. Daarna kan, als het weefsel is onderzocht, het behandelplan gemaakt worden.
OCT kan door middel van infrarood licht de verschillende lagen van de blaaswand in beeld brengen. Mogelijk kan OCT tijdens een kijkonderzoek al het tumortype bepalen en kan er sneller een behandelplan worden gemaakt.

4. Hoe verloopt het onderzoek?

Hoelang duurt het onderzoek?
Doet u mee met het onderzoek? Dan duurt dat in totaal, vanaf het geven van de toestemming tot het moment dat de uitslag van het weefselonderzoek bekend is, ca. 8 weken. De onderzoeksmetingen zelf duren slechts ca. 20 minuten.

Stap 1: bent u geschikt om mee te doen?
Uw behandelend arts heeft vastgesteld dat er één of meerdere afwijkingen in uw blaas zitten, waarvoor u gepland wordt/bent voor een operatie waarbij deze zal/zullen worden weggehaald. We willen eerst weten of u geschikt bent om mee te doen. Daarom zal de onderzoeker de afbeeldingen van de afwijking(en) in de blaas en het verslag van het kijkonderzoek beoordelen. Afhankelijk van de grootte en de locatie van de afwijking(en) in de blaas bent u wel/niet geschikt voor deelname aan dit onderzoek. De onderzoeker zal dit met u bespreken en toelichten.

Stap 2: de metingen
Tijdens de operatie zullen we 2 maal metingen met de OCT katheter uitvoeren. De voorbereiding van de operatie zal verlopen zoals bij de standaard zorg. Voordat we de afwijking(en) in de blaas weghalen zullen we onderzoeksmetingen op de afwijking(en) doen. Daarna zullen we de afwijking(en) in de blaas weghalen, dit valt onder de standaard zorg. Vervolgens zullen we nog een keer onderzoeksmetingen doen op de plek waar de afwijking(en) hebben gezeten. De OCT katheter zal in de blaas worden gebracht door het werkkanaal van het instrument dat voor de operatie wordt gebruikt om in de blaas te kijken. .

Stap 3: onderzoeken
Voor het onderzoek zijn geen extra afspraken, onderzoeken of vragenlijsten nodig.

Wat is er anders dan bij gewone zorg?
Er is bij dit onderzoek niet zoveel anders dan bij gewone zorg. Er worden tijdens de operatie 2 keer metingen gedaan van de afwijking(en) in uw blaas, de operatie zal hierdoor ca. 20 minuten langer duren. Er horen bij dit onderzoek geen extra controles of vragenlijsten. De controles na de operatie zullen enkel de gebruikelijke controle bezoeken zijn.

5. Welke afspraken maken we met u?

We willen graag dat het onderzoek goed verloopt. Daarom maken we de volgende afspraken met u:

- U neemt contact op met de onderzoeker in deze situaties:
    - U wordt in de maand na de operatie in een ziekenhuis opgenomen of behandeld.
    - U bent zwanger geworden.
    - U wilt niet meer meedoen met het onderzoek.

6. Van welke bijwerkingen, nadelige effecten of ongemakken kunt u last krijgen?

Er zijn geen te verwachten bijwerkingen, nadelige effecten of ongemakken van de metingen met de OCT katheter.

7. Wat zijn de voordelen en de nadelen als u meedoet aan het onderzoek?

Meedoen aan het onderzoek kan voordelen en nadelen hebben. Hieronder zetten we ze op een rij. Denk hier goed over na, en praat erover met anderen.
Als u meedoet aan dit onderzoek betekent het niet dat uw ziekte ervan overgaat. Maar met uw deelname helpt u mee in de zoektocht naar een betere diagnostiek van blaaskanker.

Meedoen aan het onderzoek kan deze nadelen of gevolgen hebben:

- Meedoen aan het onderzoek verlengt de operatie, en daarmee de narcose, met ca. 20 minuten.
- De OCT katheter wordt via het werkkanaal dat in de blaas zit ingebracht. Deze katheter is uitgebreid getest in een testomgeving, maar nog niet in patiënten. Een van de evaluatiemomenten van de studie is de toepasbaarheid van de katheter. Mogelijke risico’s zijn het loslaten van onderdelen van de katheter, die met een paktang uit de blaas kunnen worden verwijderd, en daardoor mogelijk schade aan de blaaswand. Deze paktang zal via het werkkanaal van het instrument dat voor de operatie wordt gebruikt om in de blaas te kijken worden ingebracht. Het risico op het loslaten van een onderdeel van de katheter is echter zeer klein.

Wilt u niet meedoen?

U beslist zelf of u meedoet aan het onderzoek. Wilt u niet meedoen? Dan krijgt u de gewone behandeling voor blaaskanker middels de operatie.

8. Wanneer stopt het onderzoek?

De onderzoeker laat het u weten als er nieuwe informatie over het onderzoek komt die belangrijk voor u is. De onderzoeker vraagt u daarna of u blijft meedoen.

In deze situaties stopt voor u het onderzoek:

- Zodra de uitslag van het weefselonderzoek bekend is.
- U bent zwanger geworden.
- U wilt zelf stoppen met het onderzoek. Dat mag op ieder moment. Meld dit dan meteen bij de onderzoeker. U hoeft er niet bij te vertellen waarom u stopt. U krijgt dan weer de gewone behandeling voor blaaskanker.
- De onderzoeker vindt het beter voor u om te stoppen.
- Een van de volgende instanties besluit dat het onderzoek moet stoppen:
    - het Amsterdam UMC,
    - de overheid, of
    - de medisch-ethische commissie die het onderzoek beoordeelt.

Wat gebeurt er als u stopt met het onderzoek?
De onderzoekers gebruiken de gegevens (bijvoorbeeld de OCT afbeeldingen) die tot het moment van stoppen zijn verzameld.

Het hele onderzoek is afgelopen als alle deelnemers klaar zijn.

9. Wat gebeurt er na het onderzoek?

Krijgt u de resultaten van het onderzoek?
Ongeveer 1 jaar nadat het onderzoek is afgerond laat de onderzoeker u weten wat de belangrijkste uitkomsten zijn van het onderzoek. Wilt u dit niet weten? Zeg dat dan tegen de onderzoeker. Hij/zij zal het u dan niet vertellen.

10. Wat doen we met uw gegevens?

Doet u mee met het onderzoek? Dan geeft u ook toestemming om uw gegevens te verzamelen, gebruiken en bewaren.

Welke gegevens bewaren we?
We bewaren deze gegevens
- uw naam
- uw geslacht
- uw geboortedatum
- gegevens over uw gezondheid

- (medische) gegevens die we tijdens het onderzoek verzamelen

Waarom verzamelen, gebruiken en bewaren we uw gegevens?
We verzamelen, gebruiken en bewaren uw gegevens om de vragen van dit onderzoek te kunnen beantwoorden. En om de resultaten te kunnen publiceren.

Hoe beschermen we uw privacy?
Om uw privacy te beschermen geven wij uw gegevens een code. Op al uw gegevens zetten we alleen deze code. De sleutel van de code bewaren we op een beveiligde plek in het ziekenhuis. Als we uw gegevens en lichaamsmateriaal verwerken, gebruiken we steeds alleen die code. Ook in rapporten en publicaties over het onderzoek kan niemand terughalen dat het over u ging.

Wie kunnen uw gegevens zien?

Sommige personen kunnen wel uw naam en andere persoonlijke gegevens zonder code inzien. Dit kunnen gegevens zijn die speciaal voor dit onderzoek zijn verzameld, maar ook gegevens uit uw medisch dossier.
Dit zijn mensen die controleren of de onderzoekers het onderzoek goed en betrouwbaar uitvoeren. Deze personen kunnen bij uw gegevens komen:

- Leden van de commissie die de veiligheid van het onderzoek in de gaten houdt.
- Een controleur die voor de opdrachtgever werkt.
- Nationale en internationale toezichthoudende autoriteiten.

Deze personen houden uw gegevens geheim. Voor inzage door deze personen vragen wij u toestemming te geven. De Inspectie Gezondheidszorg en Jeugd kan zonder uw toestemming uw gegevens inzien.

Hoelang bewaren we uw gegevens?
We bewaren uw gegevens 15 jaar in het ziekenhuis.

Mogen we uw gegevens gebruiken voor ander onderzoek?
Uw verzamelde gegevens kunnen ook van belang zijn voor ander wetenschappelijk onderzoek op het gebied van blaaskanker. Daarvoor zullen uw gegevens 15 jaar worden bewaard in het ziekenhuis. In het toestemmingformulier geeft u aan of u dit goed vindt. Geeft u geen toestemming? Dan kunt u nog steeds meedoen met dit onderzoek. U krijgt dezelfde zorg.

Wat gebeurt er bij onverwachte ontdekkingen?
Tijdens het onderzoek kunnen we toevallig iets vinden dat niet direct van belang is voor het onderzoek maar wel voor uw gezondheid. De onderzoeker neemt dan contact op met uw huisarts. U bespreekt dan met uw huisarts of specialist wat er moet gebeuren. De kosten hiervan vallen onder uw eigen zorgverzekering. U geeft met het formulier toestemming voor het informeren van uw huisarts of specialist.

Kunt u uw toestemming voor het gebruik van uw gegevens weer intrekken?
U kunt uw toestemming voor het gebruik van uw gegevens op ieder moment intrekken. Zeg dat dan tegen de onderzoeker. Maar let op: trekt u uw toestemming in, en hebben onderzoekers dan al gegevens verzameld voor een onderzoek? Dan mogen zij deze gegevens nog wel gebruiken.

Wilt u meer weten over uw privacy?

- Wilt u meer weten over uw rechten bij de verwerking van persoonsgegevens? Kijk dan op www.autoriteitpersoonsgegevens.nl.
- Heeft u vragen over uw rechten? Of heeft u een klacht over de verwerking van uw persoonsgegevens? Neem dan contact op met degene die verantwoordelijk is voor de verwerking van uw persoonsgegevens. Voor uw onderzoek is dat:
    - het Amsterdam UMC. Zie bijlage A voor contactgegevens, en website.
- Als u klachten heeft over de verwerking van uw persoonsgegevens, raden we u aan om deze eerst te bespreken met het onderzoeksteam. U kunt ook naar de Functionaris Gegevensbescherming van het Amsterdam UMC gaan. Of u dient een klacht in bij de Autoriteit Persoonsgegevens.

Waar vindt u meer informatie over het onderzoek?
Op de volgende website(s) vindt u meer informatie over het onderzoek: www.ClinicalTrials.gov. Na het onderzoek kan de website een samenvatting van de resultaten van dit onderzoek tonen. U vindt het onderzoek door te zoeken op ‘OCT and Bladder cancer’ (nummer: NCT06679920)

11. Krijgt u een vergoeding als u meedoet aan het onderzoek?

De extra testen voor het onderzoek kosten u niets. U krijgt ook geen vergoeding als u meedoet aan dit onderzoek.

12. Bent u verzekerd tijdens het onderzoek?

Voor iedereen die meedoet aan dit onderzoek is een verzekering afgesloten. De verzekering betaalt voor schade door het onderzoek. Maar niet voor alle schade. In **bijlage B** vindt u meer informatie over de verzekering en de uitzonderingen. Daar staat ook aan wie u schade kunt melden.

13. We informeren uw huisarts

De onderzoeker stuurt uw huisarts een brief om te laten weten dat u meedoet aan het onderzoek. Dit is voor uw eigen veiligheid.

14. Heeft u vragen?

Vragen over het onderzoek kunt u stellen aan het onderzoeksteam. Wilt u advies van iemand die er geen belang bij heeft? Ga dan naar de onafhankelijk deskundige, voor

contactgegevens zie bijlage A. Hij weet veel over het onderzoek, maar werkt niet mee aan dit onderzoek.
Heeft u een klacht? Bespreek dit dan met de onderzoeker of de arts die u behandelt. Wilt u dit liever niet? Ga dan naar klachtencommissie van uw ziekenhuis. In bijlage A staat waar u die kunt vinden.

15. Hoe geeft u toestemming voor het onderzoek?

U kunt eerst rustig nadenken over dit onderzoek. Daarna vertelt u de onderzoeker of u de informatie begrijpt en of u wel of niet wilt meedoen. Wilt u meedoen? Dan vult u het toestemmingsformulier in dat u bij deze informatiebrief vindt. U en de onderzoeker krijgen allebei een getekende versie van deze toestemmingsverklaring.

Dank voor uw tijd.

16. Bijlagen bij deze informatie

A. Contactgegevens

B. Informatie over de verzekering

C. Toestemmingsformulier

Bijlage A: contactgegevens voor Amsterdam UMC

Hoofdonderzoeker: J.R. Oddens, uroloog
Telefoonnummer: ***
Mail: ***

Onderzoeksarts: drs. M.J. Remmelink, arts-onderzoeker
Telefoonnummer: ***
Mail: ***

Onafhankelijk arts: dr. H.J.R. van der Horst, uroloog
Telefoonnummer: ***
Mail: ***

Klachten: Patiëntenservice Zorgsupport locatie VUMc
Mail: ***

Functionaris voor de Gegevensbescherming van de instelling:
Mail: ***

Bijlage B: informatie over de verzekering

Het Amsterdam UMC heeft een verzekering afgesloten voor iedereen die meedoet aan het onderzoek. De verzekering betaalt de schade die u heeft doordat u aan het onderzoek meedeed. Het gaat om schade die u krijgt tijdens het onderzoek, of binnen 4 jaar na het einde van uw deelname aan het onderzoek. U moet schade binnen 4 jaar melden bij de verzekeraar.

Heeft u schade door het onderzoek? Meld dit dan bij Patiëntenservice Zorgsupport locatie VUMc, zij zullen de claim doorgeven aan de verzekeraar:

De verzekeraar van het onderzoek is:	
Naam:	Centramed B.A.
Adres:	Postbus 7374<br>2701 AJ Zoetermeer
Telefoonnummer:	070 3017070
E-mail:	info@centramed.nl
Polisnummer:	624.529.204

De verzekering biedt een dekking van €650.000 per proefpersoon met een maximum van € 5.000.000 voor het hele onderzoek en € 7.500.000 voor schade ten gevolge van medisch-wetenschappelijk onderzoek die per verzekeringsjaar wordt gemeld.

Let op: de verzekering dekt de volgende schade **niet**:

- Schade door een risico waarover we u informatie hebben gegeven in deze brief. Maar dit geldt niet als het risico groter bleek te zijn dan we van tevoren dachten. Of als het risico heel onwaarschijnlijk was.
- Schade aan uw gezondheid die ook zou zijn ontstaan als u niet aan het onderzoek had meegedaan.
- Schade die ontstaat doordat u aanwijzingen of instructies niet of niet goed opvolgde.
- Schade aan de gezondheid van uw kinderen of kleinkinderen.
- Schade door een behandelmethode die al bestaat. Of door onderzoek naar een behandelmethode die al bestaat.

Deze bepalingen staan in het 'Besluit verplichte verzekering bij medisch-wetenschappelijk onderzoek met mensen 2015'. Dit besluit staat in de Wettenbank van de overheid (https://wetten.overheid.nl).

Bijlage C: toestemmingsformulier proefpersoon

Behorende bij

Nieuwe beeldvormende techniek voor blaastumoren tijdens blaaskijkonderzoek.

- Ik heb de informatiebrief gelezen. Ook kon ik vragen stellen. Mijn vragen zijn goed genoeg beantwoord. Ik had genoeg tijd om te beslissen of ik meedoe.
- Ik weet dat meedoen vrijwillig is. Ook weet ik dat ik op ieder moment kan beslissen om toch niet mee te doen met het onderzoek. Of om ermee te stoppen. Ik hoef dan niet te zeggen waarom ik wil stoppen.
- Ik geef de onderzoeker toestemming om mijn huisarts die mij behandelt te laten weten dat ik meedoe aan dit onderzoek.
- Ik geef de onderzoeker toestemming om mijn huisarts of specialist informatie te geven over onverwachte bevindingen uit het onderzoek die van belang zijn voor mijn gezondheid.
- Ik geef de onderzoekers toestemming om mijn gegevens te verzamelen en gebruiken. De onderzoekers doen dit alleen om de onderzoeksvraag van dit onderzoek te beantwoorden.
- Ik weet dat voor de controle van het onderzoek sommige mensen al mijn gegevens kunnen inzien. Die mensen staan in deze informatiebrief. Ik geef deze mensen toestemming om mijn gegevens in te zien voor deze controle.

- Wilt u in de tabel hieronder ja of nee aankruisen?

Ik geef toestemming om mijn gegevens te bewaren om dit te gebruiken voor ander onderzoek, zoals in de informatiebrief staat.	Ja ☐	Nee☐
Ik geef toestemming om mij eventueel na dit onderzoek te vragen of ik wil meedoen met een vervolgonderzoek.	Ja ☐	Nee☐

- Ik wil meedoen aan dit onderzoek.

Mijn naam is (proefpersoon): ………………………………..

Handtekening: ………………………   Datum : __ / __ / __

Ik verklaar dat ik deze proefpersoon volledig heb geïnformeerd over het genoemde onderzoek.

Wordt er tijdens het onderzoek informatie bekend die die de toestemming van de proefpersoon kan beïnvloeden? Dan laat ik dit op tijd weten aan deze proefpersoon.

Naam onderzoeker (of diens vertegenwoordiger):……………………………….

Handtekening:………………………   Datum: __ / __ / __

De proefpersoon krijgt een volledige informatiebrief mee, samen met een getekende versie van het toestemmingsformulier.
